# Estimating dengue disease and economic burden to inform municipal-level policymakers: Method for a pragmatic city-level observational cohort study

**DOI:** 10.12688/gatesopenres.15015.1

**Published:** 2024-01-08

**Authors:** Nandyan N. Wilastonegoro, Sri Andriani, Perigrinus H. Sebong, Priya Agarwal-Harding, Donald S. Shepard

**Affiliations:** 1 Faculty of Medicine, Public Health, and Nursing, Gadjah Mada University, Yogyakarta, Special Region of Yogyakarta, 55281, Indonesia; 2 Health Department, Government of Semarang City, Semarang, Central Java, 50249, Indonesia; 3 Faculty of Medicine, Soegijapranata Catholic University, Semarang, Central Java, 50215, Indonesia; 4 The Heller School for Social Policy and Management, Brandeis University, Waltham, Massachusetts, 02453, USA

**Keywords:** cost, dengue, economic burden, Indonesia, pragmatic, presenteeism, protocol, surveillance

## Abstract

**Background:**

Recent trials have confirmed the effectiveness of promising dengue control technologies – two vaccines, and *Wolbachia*. These would generally be applied at the municipal level. To balance health needs and resource constraints, local officials need affordable, timely, and accurate data. Building on our previous work in Mexico, Indonesia, and Thailand, we developed a streamlined prospective method to estimate dengue burden at the municipal level quickly, accurately, and efficiently.

**Method:**

The method entails enrolling and repeatedly interviewing 100 patients with laboratory-confirmed dengue. They will be selected after screening and testing about 1,000 patients with clinical dengue. The method will capture both acute and chronic effects relating to disease, economic burden, and psychological impacts (presenteeism). The total time requirements are 1.5 years, comprised of 0.25 years for planning and approvals, 1 year for data collection (a full dengue cycle), and 0 .25 years for data cleaning and analysis. A collaboration with municipal and academic colleagues in the city of Semarang, Central Java, Indonesia shows how the method could be readily applied in Indonesia’s eighth largest city (population 1.8 million).

**Conclusions:**

Many surveillance studies gather only information on numbers of cases. This proposed method will provide a comprehensive picture of the dengue burden to the health system, payers, and households at the local level.

## Introduction

### Overview of dengue

Dengue, classified by the World Health Organization as a neglected tropical disease, has substantial health and economic impacts on healthcare systems in most tropical and subtropical countries worldwide ^1– 4^. As a disease prone to pandemics, the occurrence of dengue has seen a dramatic increase over the last five decades, leading to an estimated 41,000 deaths and nearly US$9 billion in annual economic costs ^2, 5^. Although almost half of the global population resides in dengue-endemic regions, the Asian region bears more than 70% of the disease burden ^6^.

Dengue is caused by the dengue virus (DENV), which has four strains: DENV-1, DENV-2, DENV-3, and DENV-4. It is transmitted through the bite of *Aedes* mosquitoes, primarily *Aedes aegypti* and *Aedes albopictus* ^7^. DENV infection can manifest in various ways, ranging from asymptomatic or mild flu-like symptoms known as dengue fever (DF), to more severe forms such as dengue hemorrhagic fever (DHF) and dengue shock syndrome (DSS). A symptomatic dengue infection follows a three-phase pattern within the first 10 days after onset: the febrile phase, the critical phase, and the recovery phase ^8, 9^. In the febrile phase, the patient experiences viremia, leading to high fever, hemoconcentration, and dehydration, which may last 2–7 days with a minimum temperature of 38.5°C. During the critical phase, occurring around 3–7 days from onset, there may be hemorrhagic manifestations or dengue shock due to plasma leakage and hemoconcentration, affecting various organs. The final stage, the recovery or convalescence phase, lasting approximately 48–72 hours, may present complications due to fluid overload and reabsorption issues.

However, some dengue patients may also experience a persistent phase, characterized by symptoms that result in a decreased capacity to work, such as fatigue, depression, and weight loss, which may persist for several weeks following the recovery stage ^9– 11^. The incidence of persistent dengue is largely unknown. A limited number of studies have aimed to estimate the incidence, although a study in Malaysia estimated that between 5–10% of patients who experience symptomatic dengue may experience the persistent phase, with these patients experiencing adverse effects on their quality of life well beyond the febrile phase ^12^. Another recent study conducted in Morelos, Mexico found that 55.7% of patients included in their study experienced persistent symptoms one month after the onset of symptoms ^13^ and a study in Brazil estimated that 20% of patients experienced persistent symptoms 60 days after the onset of illness ^14^. A systematic analysis conducted by Zeng *et al.* (2018) also estimated that 34 % of dengue episodes experienced persistent symptoms, which contributed to over half of the disability-adjusted life year (DALY) burden per episode for both ambulatory and hospitalized cases ^15^. Thus, including the persistent phase in analyses of the economic and societal burden of a dengue episode is essential in understanding the complete burden of the disease and informing the targeting of dengue-specific public health interventions.

### Dengue control technologies

Complicating the public health challenge, prior to the past decade, most dengue prevention and control measures had little evidence of efficacy or were difficult to implement and hard to sustain. Fortunately, within the past decade, two new promising technologies have emerged for addressing dengue—(1) several tetravalent dengue vaccines and (2) the release of virus-blocking *Wolbachia-*infected mosquitoes. Both technologies have been tested through randomized control trial (RCT) designs and were found to be efficacious in preventing dengue. In terms of the vaccine candidates, the two-dose Takeda vaccine (TAK-003) is 61.2% efficacious according to the latest (54-month) data, and the newest (Bhutantan) vaccine, requiring only a single dose, is 79.6% efficacious at 24 months. These vaccines have the advantage over the previously approved Sanofi vaccine (Dengvaxia), which required testing or a detailed medical history to rule out administering it to non-immune candidates ^16^. *Wolbachia* proved 77.1% efficacious against symptomatic dengue in a cluster RCT in Yogyakarta, Indonesia ^17^.

Recent studies on the cost-effectiveness of *Wolbachia*-infected mosquitoes in Indonesia found that use in high-density urban areas is expected to be highly cost-effective and could potentially even be cost-saving ^18, 19^.

While both approaches hold promise for addressing dengue, given the limited availability of donor financing for global dengue prevention and treatment, countries will likely have to predominantly fund the implementation of these technologies through domestic budgets. To aid governments in effectively targeting dengue interventions and prioritizing prevention and treatment among various health concerns, comprehensive and country-specific studies on the economic costs and burden of dengue are imperative. Furthermore, studies that can address variations in dengue burden based on diverse social demographics and dengue serotypes are crucial to inform tailored dengue control strategies for distinct population groups.

### Gaps on quality of life

Despite numerous epidemiologic studies, there remains a scarcity of economic studies and empirical investigations measuring the impact of dengue on quality of life. Notably, studies examining the effects of a complete dengue episode, including cases with persistent symptoms, are particularly lacking. Our review of the existing literature on dengue and associated costs reveals that most studies (1) have a limited focus on the acute phases of the disease, (2) exclude children from their study designs, (3) employ only crude impact measures, and (4) have not been able to estimate the frequency, intensity, or duration of persistent symptoms.

### Gaps on persistent dengue

Tiga *et al.* ^11^ have summarized selected studies on persistent dengue. While most studies describe general signs and symptoms of persistent dengue, including asthenia, sleepiness, lethargy, myalgia, arthralgia, decreased appetite, irritability, headaches after physical exertion, and hair loss ^20^, only a few studies have examined specific endpoints, such as lower platelet count (thrombocytopenia) ^21^, dysgeusia, myositis ^22^, decreased visual acuity, cardiomyopathy, neurological defects, and elevated liver transaminases ^23^. Persistent symptoms were associated with females, persons of older age, and disease severity in some studies ^12, 24– 28^. However, studies on risk factors are limited, and, to date, there is no understanding of the physio-pathological mechanisms of persistent symptoms following dengue infection. Persistent symptoms have also been shown to have negative effects on patients’ quality of life ^12, 15, 29^, productivity, and generate additional medical expenses ^10^, thereby imposing additional economic costs beyond the acute phase.

### Gaps on costs

Indonesia has produced two studies of the cost of a dengue episode ^30, 31^. While both were useful, they did not necessarily generate a representative sample of dengue episodes nor include persistent dengue. Due to the large burden of disease imposed by persistent dengue, it is very likely that current estimates of disease and economic burden are substantially underestimated, especially if persistent symptoms affect a non-negligible share of the population ^15^. Based on a systematic review of literature focused on persistent symptoms that may reduce an individual’s capacity to work, members of this research team estimated additional economic costs that persistent dengue may impose in Mexico. Persistent dengue resulted in a 13% increase in economic costs of dengue in Mexico, due mainly to productivity losses, and a 43% increase in DALYs over previous estimates ^10^. This is a substantial contribution to the overall burden of dengue in the country and suggests that greater understanding of persistent dengue as a part of the overall burden of dengue will also be essential in other countries, especially those that are weighing various dengue-specific public health interventions amongst a myriad of other public health priorities.

Additionally, while most existing costing studies may focus on the impacts of work absenteeism (costs associated with missed scheduled work or school from dengue illness) in their estimates of dengue burden, the impact of presenteeism, defined as reduced productivity resulting from presenting to work or school when feeling poorly or still being ill, has generally received little or no attention despite studies showing large productivity losses at work from a reduced capacity to work due to illness ^32, 33^. To date, no published study has examined the loss of presenteeism in dengue specifically and its relationship to overall economic burden of dengue.

While there have been a few studies on the economic cost of dengue, they tend to be incomplete for not capturing the impact on presenteeism and long-term health consequences. To provide a more complete picture of the burden of dengue in health and economic terms, we developed this episode-based method. The primary objective is to generate a more complete understanding of the complete burden of an episode of dengue, including the cost to the health system, to families, and loss in quality of life (including decrease in presenteeism) of a complete episode of dengue. This loss should include both acute and persistent phases of an episode. The episode-based method addresses the following components:

(1) studying dengue episodes over a longer time period, including persistent symptoms up to six months after the onset of the disease;

(2) including time-sensitive measures of economic impact, including work presenteeism;

(3) including a broader range of economic cost data, including all costs related to symptomatic dengue regardless of who pays for costs or receives benefits (all household, direct medical and non-medical, and indirect costs);

(4) making use of robust laboratory measures to identify dengue cases as well as dengue serotypes;

(5) including non-medical cases, based on self-report.

This method includes probing questions to identify probable dengue cases within the households of enrolled patients and ask about their health-seeking behavior and the direct and indirect cost of the probable dengue. The proposed method will enroll patients from diverse types of health facilities in both public and private sectors. This method suggests enrolling patients at hospitals (both public and private), public health centers, and private clinics. This will allow for more accurate representation of the dengue burden in the study location and extrapolation to other similar contexts, as well as enable the study to examine the impacts of the kind of facility a patient presents to on the burden of dengue. The method includes point-of-care rapid testing with dual rapid diagnostic test which tests both NS1 and IgG/IgM. Only patients who test positive on NS1 and/or IgM on this rapid test will be invited to enroll. The method will optionally add RT-PCR testing to examine whether and how persistence may vary by dengue serotype on the history of past infections. This will add useful information for targeting of vaccines (such as by serotype and/or demographics) and informing clinical management. Patient blood samples used for RT-PCR testing could also be used to determine genetic sequencing of dengue at the sites which will add additional information on differences in dengue incidence and burden by geographic areas. This optional component does, however, add the cost of the added laboratory testing and the logistical burden of collecting and transporting the specimens to a laboratory. Since the RT-PCR testing is part of the enrollment process, it is not time sensitive. This means that the specimens could be transported and tested on a batch basis, thereby reducing the cost.

This method of dengue disease and economic surveillance will benefit the country’s researchers, policymakers, and donors in informing scientific, data-driven decisions about dengue control. It will also help inform more accurate cost-benefit and cost-effectiveness analyses for *Wolbachia* technology, dengue vaccines, and other dengue control interventions in the implemented region and around the world.

This method seeks to evaluate the cost and quality of life impact of dengue in both acute and persistent phases. To this end, the aim is to address the following specific objectives and questions in the four areas of (1) epidemiology, (2) persistent dengue, (3) quality of life, and (4) economic burden. Altogether, it plans to answer 13 questions across four domains.

### Specific questions to be addressed

1. Epidemiology

1.1. How do cases identified through intensive surveillance compare to those routinely reported to the national Ministry of Health?

1.2. Which months are categorized as the highest and lowest season of having dengue?

1.3. What is the proportion of confirmed dengue cases among probable dengue cases?

1.4. What is the level of under-reporting of medically attended dengue?

1.5. What is the extent of small-scale geographical clustering of reported dengue cases in the city/district linked to an index beyond what will occur by chance?

2. Persistent dengue

2.1. What is the burden of persistent dengue when it occurs?

2.2. What is the rate of persistent dengue?

2.3. What variables predict persistent or chronic dengue, considering a range of factors: demographics, prior dengue infection, dengue serotype, the lag between the onset of fever and diagnosis, type of management, socio-economic characteristics?

3. Quality of life

3.1. What are the symptoms of acute and persistent dengue?

3.2. What challenges to health-related quality of life persist after recovery from the acute phase?

3.3. What is the impact of acute and persistent phases of dengue on presenteeism?

4. Economic burden

4.1. What is the economic burden of dengue per episode, including burdens due to the acute phase, persistent symptoms, reduction in presenteeism, and cases treated in non-medical settings?

4.2. What is the relationship between socioeconomic status on the economic burden of dengue?

### Need for municipal-level knowledge

Control strategies must often be financed and/or implemented at a city level, especially *Wolbachia*. Vaccination often proceeds through local health posts. Because dengue epidemiology varies, each municipality may have a different disease profile and potentially a different control strategy. This method has been deliberately created with a moderate sample (100 patients interviewed) to keep the time and cost limited.

## Proposed method

### Overview

The method is based on a prospective-cohort of patients with symptomatic dengue infection over a three-year time-period. The method includes one full dengue season and one partial dengue season in the first year.

This method will recruit patients at selected inpatient and outpatient health facilities at the implementation city/district as participants according to clinical diagnosis. As the main goal is descriptive, rather than hypothesis testing, the larger and more diverse the sample, the greater the precision and the more representative the participants. Based on the authors’ past experience, the proposed sample of 100 patients with a 1.5-year overall time period generates sufficient data for action while being quick enough to be operationally relevant.

### Illustrative application: Semarang City, Indonesia

**Figure 1.**
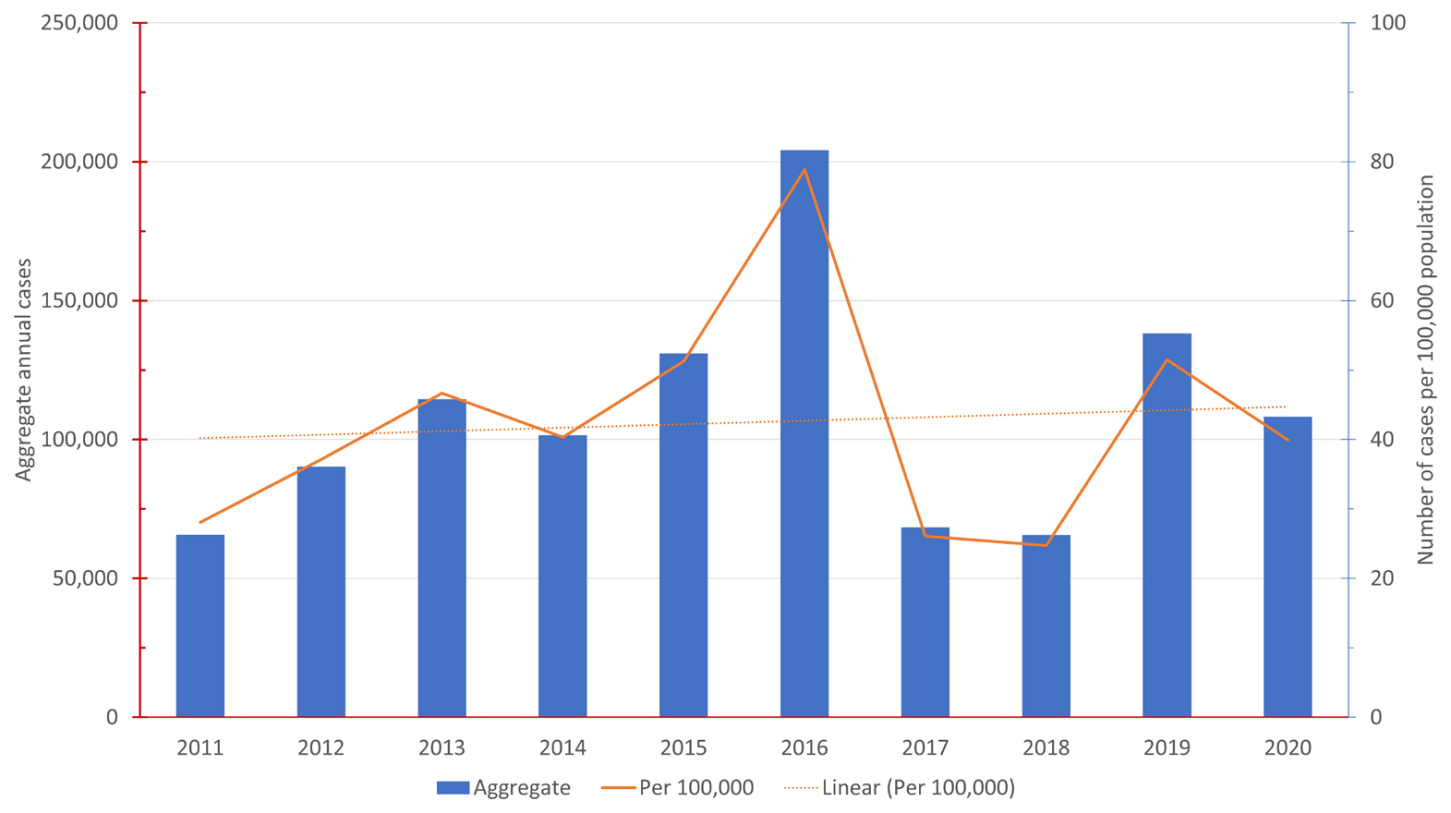
Annual Reported Dengue Hemorrhagic Fever (DHF) Cases 2011 – 2020.

Indonesia is the most populous country in dengue-endemic South-East Asia and has consistently ranked as the country with the most dengue cases annually ^1– 4, 34^. While multiple studies have estimated the disease burden associated with dengue in Indonesia using a variety of data sources and methods ^2, 3, 6, 35– 37^, current dengue surveillance in the country is limited, with only DHF cases being collected and reported at the national level. Members of this research team estimated that there were approximately 7.8 million symptomatic dengue cases in Indonesia in 2015, associated with a loss of 332,865 disability-adjusted life years (DALYs) ^38^. It was estimated that most of the dengue burden in that study was due to non-severe cases that did not seek treatment or were challenging to diagnose in outpatient settings, and the burden was highly concentrated in just a few cities. This result highlights the need for more complete dengue surveillance systems in the country that also include data on less severe DF cases. Figure 1 shows the number of annual reported DHF dengue cases in Indonesia from 2011–2020 both in aggregate and the rate per 100,000 population.

**Figure 2.**
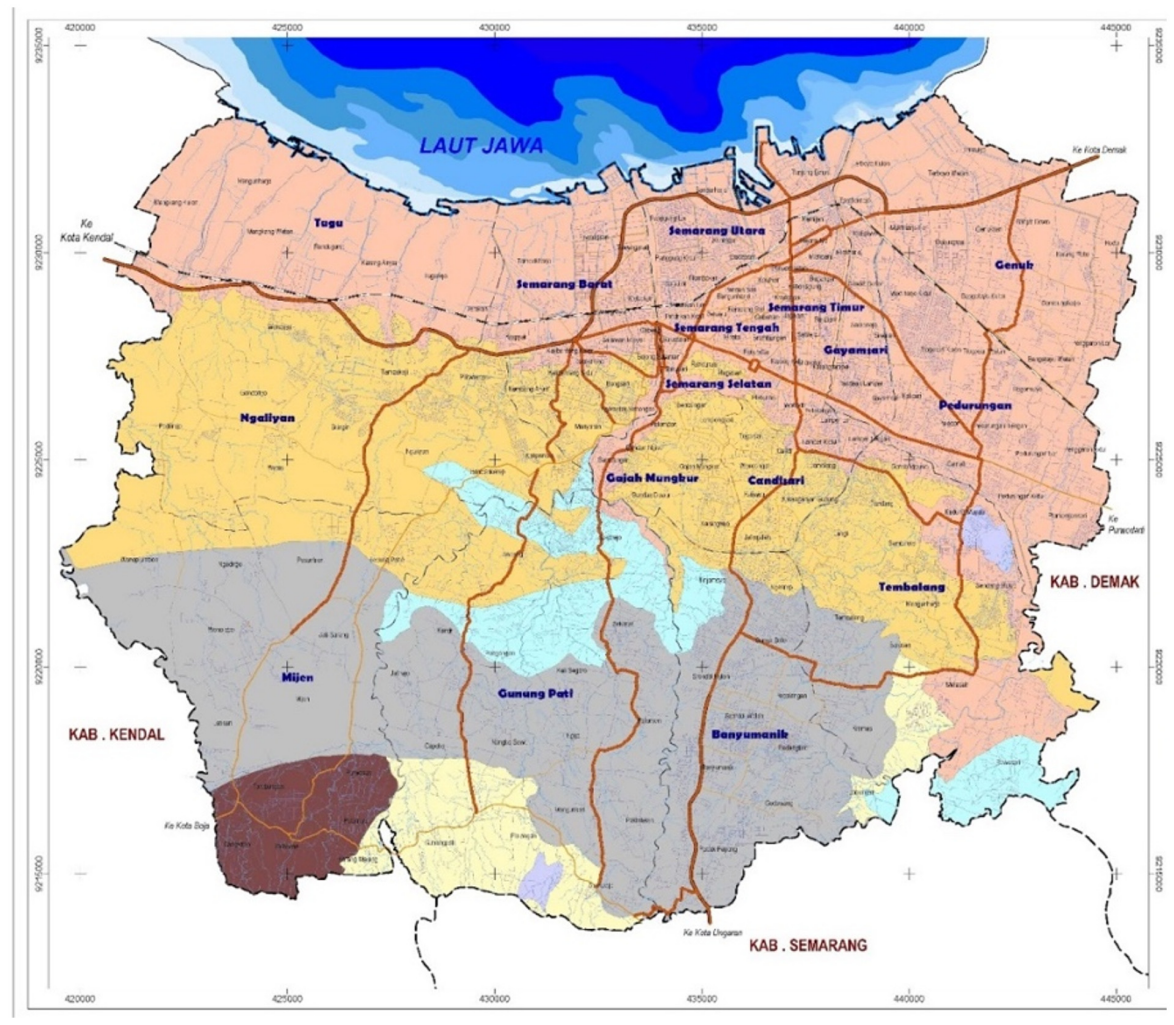
Map of Semarang City. Source: Semarang City Planning and Development Board’s website: https://bappedasemarang.wordpress.com/asd/.

Semarang City is a moderately populous city within Central Java Province, Indonesia. We selected it to illustrate how this method could be implemented due to its strong sub-national surveillance system; well-qualified City Health Department; and proximity to Yogyakarta, where the authors recently published analyses of dengue burden ^17, 31^). Semarang City ranks eighth in size of cities in Indonesia by population size ^39^, making it both moderate and manageable ( Figure 2). With no other interventions for dengue ongoing nor currently planned in the area, results will not be affected by confounding from other programs.

Administratively, the city is divided into 16 districts and 177 sub-districts. It lies in the northern-central part of Java Island and is bordered by the Java Sea on the North, Demak Regency on the East, Semarang Regency on the South, and Kendal Regency on the West. With an estimated population of 1,668,578 people (2019) and a land area of 373.7 km ^2^, Semarang City has a population density of approximately 4,855 people per km ^2^ ^40^.

### Dengue surveillance in Semarang

**Figure 3.**
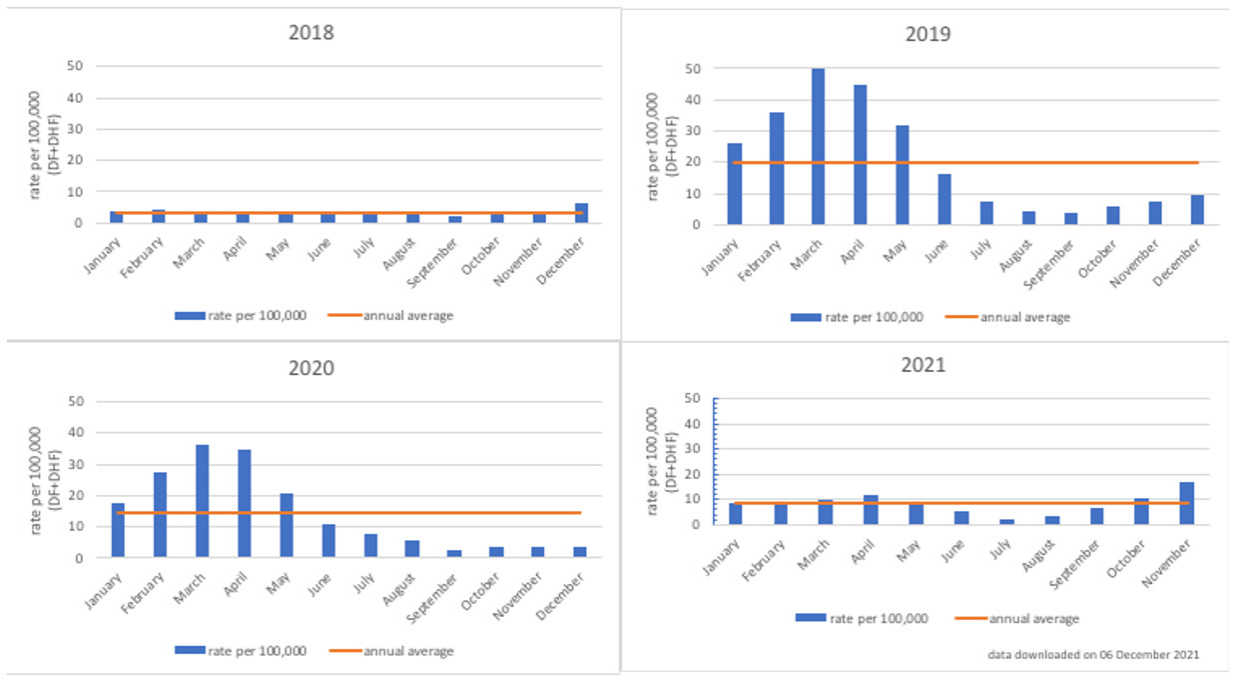
Rate of hospitalized dengue cases per 100,000 population in Semarang City, 2018–2021. Source: Semarang City Health Department.

Since dengue was initially recognized in Surabaya and Jakarta, Indonesia in 1968, dengue has also become a concern in Semarang City. Data from an integrated web-based dengue reporting system to monitor dengue prevalence and inform policy decisions developed by the Semarang City Health Department, *Tunggaldara* (an abbreviation of “ *Bersatu Tanggulangi Demam Berdarah*” in Bahasa Indonesia, translated as “to fight dengue haemorrhagic fever together”), was used to calculate the annual average of hospitalized dengue cases per 100,000 population from 2018 to 2020 ( Figure 3) ^41^. The average annual number of dengue cases (DHF only) in Semarang during this period per 100,000 population was 17.23 cases. As was shown in Figure 1, the national average is approximately 42.2 annual dengue cases per 10,000 population.

With high rates of asymptomatic infection and symptoms that are common with other infectious diseases, such as chikungunya, Zika, yellow fever, and COVID-19 ^42, 43^, estimations of the dengue burden are still uncertain. In 2016, the Indonesian government launched a “Healthy Indonesia Program with Family Approach”, strengthening the government’s commitment to addressing communicable diseases, such as dengue and malaria ^44^. In the midst of COVID-19, such a commitment is even more paramount. To achieve it will require more accurate and contemporary estimates of the burden of these diseases, including dengue, to target and quantify the benefits of any DENV control strategy. Drawing from the city's surveillance system, which also includes hospitalized DF cases, the average annual number of total dengue cases (DF and DHF) in Semarang City per 100,000 population was 153 cases.

Of the officially reported cases from Semarang City Health Department, from 2018 to 2020, almost all the cases were DF (87.74%), with the remaining being DHF (12.26%). These data show that the cases reported by the City Health Department, which include DF, are more comprehensive than those of the National Ministry of Health (MOH), which tally only DHF cases. The strength of this surveillance system in Semarang City provides the method implementation with a strong source with which to compare epidemiological findings. However, it should be noted that the Semarang City reporting system also officially reports only cases that have been hospitalized. It may therefore still underestimate the burden, which should also include outpatient cases. Capturing information on both hospitalized and outpatient cases is an objective of this proposed method. Figure 3 shows that the annual pattern of dengue cases in Semarang City is like the national pattern of cases, with a concentration of dengue cases in the first six months of the year.

**Figure 4.**
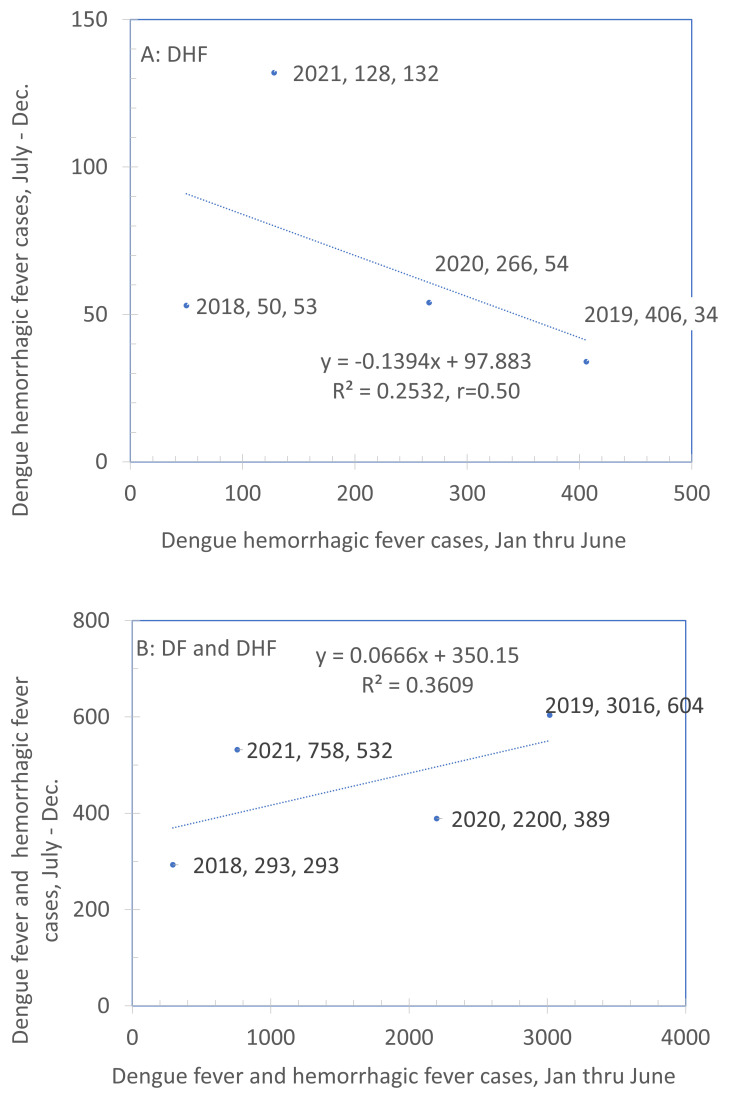
Comparison of rate of DHF cases between the first and second halves of the year (2011–2020). Note: The legend for each point indicates the year, the X-value and the Y-value. Source: Sub-Directorate of Arbovirus, Ministry of Health Indonesia.

We sought to determine whether we could use predictability in numbers of dengue cases between the first and second half of a year for planning recruitment. Figure 4 plots the rate of cases in the second half of the calendar year against those in the first half for DHF (panel A) and DHF and DF combined (panel B). We hypothesized that years in which the dengue burden was low from January to June also had lower dengue burdens in the second half of the year (July to December) and years in which dengue burden was high in the first half of the year showed a similar pattern in the second half of the year. If this were a strong, highly consistent relationship, we would be able to use surveillance data from the first half of the year to plan efforts for the second half. However, these data suggest that this is not possible. With a sample of only four years, neither association is statistically significant. Furthermore, they differ in direction with a negative association in Panel A and a positive one in Panel B. Overall, the scatter is considerable. These results show that the pattern between the first and second half of the year at the level of a municipality is highly variable. Therefore, it cannot be used for guiding efforts for the subsequent recruitment. An alternative approach, described below, will be used instead.

### Establishing the surveillance sites

Patient recruitment will be divided equally between patients seen in inpatient and outpatient health facilities in Semarang City, Indonesia with a laboratory confirmed dengue diagnosis. Public health centers (puskesmas) are the lowest level of the health system and responsible for investigating dengue cases, monitoring house index of mosquito larvae, performing fumigation, and establishing public campaigns to prevent dengue at the household level, or private clinics, according to the laboratory confirmed case definition. Outpatient dengue cases are patients who visit clinics (in hospitals, puskesmas, or private clinics) and are diagnosed as having a dengue case by the same confirmed case definition.

Several health facilities will be selected within Semarang City, drawing from facilities with the highest dengue burden (hospitals, puskesmas, or private clinics). The method design will provide a representative sample of dengue patients at a variety of facilities, while also being efficient with time and resources and generating evidence as quickly as possible.

***Hospitals:*** From the available dengue data shown from the *Tunggaldara* website, there were ten hospitals with the highest number of dengue inpatient care cases from 2018 until 2020. Together, these facilities make up almost 78% of the burden of dengue inpatient cases in these three years and include both public and private hospitals (three public and seven private). These ten hospitals will be the potential representatives for sample collection sites (see Table 1).

**Table 1.** Top ten hospitals in order from highest to lowest aggregate inpatient DF and DHF cases in Semarang City, 2018–2020.

Name of Health Facility	Ownership	Hospital level	Number of staff employed *				Dengue cases		
			GP	I	P	LA	Average # DF and DHF aggregate cases per year	%	Cumulative %
1. KRMT Wongsonegoro Hospital	City Government	B	27	4	4	33	512.33	17.66%	17.66%
2. Telogorejo Hospital	Private Institution	B	26	18	9	30	381.00	13.13%	30.79%
3. Panti Wilasa Citarum Hospital	Private Institution	B	30	11	5	10	316.00	10.89%	41.68%
4. Elisabeth Hospital	Private Institution	B	41	20	13	34	258.00	8.89%	50.57%
5. Panti Wilasa Dr. Cipto Hospital	Private Institution	C	19	3	1	12	227.00	7.82%	58.40%
6. Prof. Awalaoeddin Djamin Hospital	Provincial Police	C	6	5	3	10	147.00	5.07%	63.46%
7. William Booth Hospital	Private Institution	C	11	4	3	9	130.33	4.49%	76.55%
8. Tugurejo Hospital	Provincial Government	B	29	7	4	30	124.67	4.30%	67.76%
9. Roemani Hospital	Private Institution	C	22	4	1	10	124.67	4.30%	72.05%
10. Columbia Asia Hospital	Private Institution	B	18	10	7	11	100.33	3.46%	80.00%
**TOTAL (average year)**							2321.33		

*DF = Dengue fever, DHF = Dengue hemorrhagic fever, GP = General Practitioner, I = Internist, P = Pediatrician, LA = Laboratory analyst. Source: Semarang City Health Senarang City Dengue Surveillance System and Human Resource Department Ministry of Health website: 41.

***Puskesmas:*** There are 37 total puskesmas within Semarang City, including 12 facilities with inpatient facilities. Those puskesmas with inpatient services will likely have a higher volume of dengue patients and also have the capacity to conduct dengue clinical testing on patients. Outpatients who test positive for dengue will be admitted to the inpatient facilities at these puskesmas. This sample will not include puskesmas with only outpatient facilities, as it is expected that sampling patients from all 37 puskesmas would be both cumbersome and expensive (see Table 2).

**Table 2.** Description of Puskesmas and Private Clinics Included in Study Sample.

Name of Health Facility	Type	District	Number of Staff Employed		
			General Practitioner	Nurse	Laboratory Analyst
Public Facilities					
1. Banget Ayu	Puskesmas	Genuk	5	6	3
2. Halmahera	Puskesmas	Semarang Timur	5	7	3
3. Karangdoro	Puskesmas	Semarang Timur	4	4	3
4. Mangkang	Puskesmas	Tugu	4	8	3
5. Ngaliyan	Puskesmas	Ngaliyan	2	8	3
6. Mijen	Puskesmas	Mijen	4	9	3
7. Karangmalang	Puskesmas	Mijen	3	9	2
8. Gunungpati	Puskesmas	Gunungpati	4	8	2
9. Srondol	Puskesmas	Banyumanik	5	6	2
10. Ngesrep	Puskesmas	Banyumanik	4	6	2
11. Rowosari	Puskesmas	Tembalang	2	6	2
12. Telogosari Kulon	Puskesmas	Pedurungan	3	11	3
Average			3.8	7.3	2.6
Private Facilities					
1. Mitra Kita	Private Clinic	Semarang Barat	NR*	NR	NR
2. Graha Sifa	Private Clinic	Gunungpati	NR	NR	NR

***Private clinics:*** Two private clinics have been identified as falling within the top 30 health facilities in terms of aggregate dengue cases from 2018 to 2020. Both facilities will be included in the sample. These facilities both regularly report statistics to Semarang City Health Department and, like the selected puskesmas, also have lab facilities and inpatient services.

**Figure 5.**
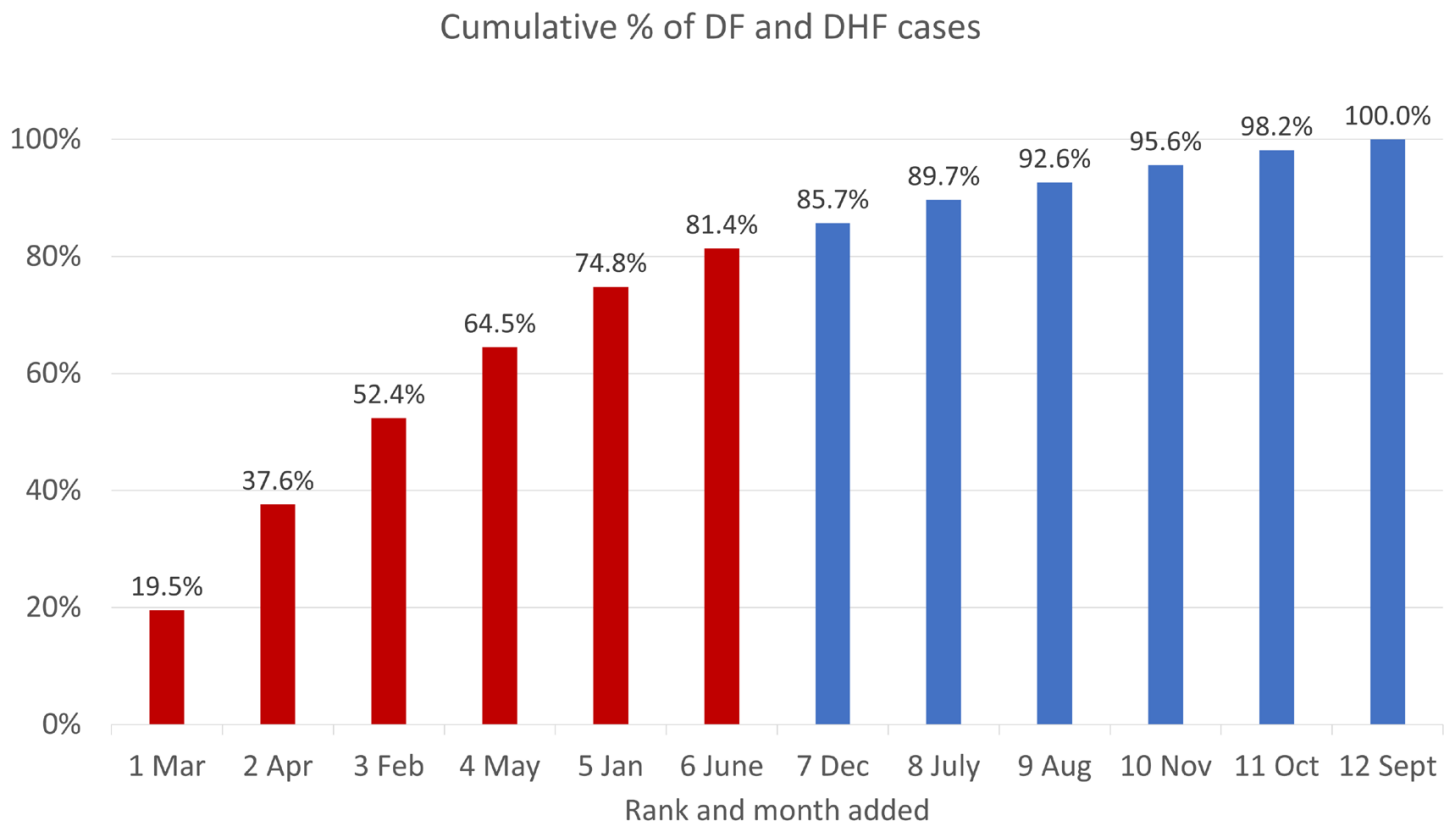
Monthly cumulative percentage of dengue cases in Semarang City, 2018–2020. Source: Semarang City Health Department Dengue Surveillance System. Note: Months in the first half of the year (higher incidence rates) are in red and those in the second half of the year (lower rates) are in red.

Drawing from the Semarang City Surveillance system, Figure 5 shows the cumulative number of hospitalized dengue cases in Semarang City from 2018 to 2020, sorted by months with the lowest to highest burden of cases. March made the highest contribution to cumulative cases (19.5%) and September had the lowest (1.8%). Like the pattern of DENV at the national level, dengue cases appear to be higher during the first half of a year, with 81.4% of cases occurring during the first semester. Patient enrollment for this study is therefore expected to follow the same pattern.

### Defining a dengue case

***Probable dengue case definition.*** A probable dengue case will be defined as a probable DENV infection in which the infected person visits a health center or hospital to receive medical care. A probable DENV infection will be diagnosed clinically, based upon fever and physical examination by physicians or other clinical staff members at health facilities, as well as from results from routine laboratory testing, such as complete blood count (CBC). This will include the patient having a history of recent fever of 38 ^o^C or higher, as well as one or more of the following: headache, retro-orbital pain, myalgia, arthralgia/bone pain, rash, hemorrhagic manifestations, leukopenia (WBC < 5000 cells/mm3), thrombocytopenia (platelet count < 150,000 cells/mm ^3^), and rising hematocrit (5–10%). Additionally, these criteria should follow the diagnostic standards within the country for DENV, based on epidemiologic information on the course of a dengue illness, and will, therefore, be relevant to epidemiological data in the country.

***Confirmed dengue case definition.*** A probable dengue patient who tests positive for either IgM or NS-1 Ag within the NS1/IgM/IgG RDTs will be considered as having a confirmed dengue episode and will be eligible for inclusion. The enrollment process is described below in the section entitled “Enrollment procedures.” These tests require a small blood sample which can be taken from routine complete blood count (CBC) testing, as part of standard diagnostic procedures for patients presenting to health facilities with fever. Therefore, these tests would not impose too much of an additional burden on patients and clinicians, are not unpleasant, can be carried out very quickly, and will provide useful information to the patient and clinician.

Under the optional component, a patient who tests positive for either or both tests will be asked to undergo an additional RT-PCR test to confirm dengue diagnosis. The patient who tests negative on the RT-PCR test will additionally be tested with an NS-1 ELISA test to capture cases that may have resulted in a false negative RT-PCR test. Together, these tests will allow researchers to determine virologically confirmed dengue cases (VCD) and provide additional useful data on dengue serotype and past dengue infections.

These tests require a 3 ml blood sample, which can also be performed using the same sample routinely collected when the patient initially presents to the hospital for completing the CBC test. As a result, this would impose very little additional burden to health facility staff and patients, and will contribute to the feasibility of this surveillance. Patients who receive negative NS-1 Ag and IgM tests but test positive for IgG in the NS1/IgM/IgG RDTs will be considered as having a probable secondary case or a past infection (non-conclusive result) and will be excluded.

***Randomly selecting cases to invite into the study.*** Our method requires an approach to select patients at random to join our prospective cohort. This step is complicated by the variability in numbers of eligible patients among sites, months and specific days. To illustrate, suppose that we had four sites and wished to enroll a quarter of our target sample from each site. Suppose there were one staff member responsible for patient enrollment. We will create cycles among the four sites, where each cycle puts the sites in a random order. To illustrate, suppose the sites were labeled A, B, C, D. Suppose the first cycle’s random order were A C B D, and the second cycle’s was D A C B. The data collection agent will pick a random day of the week and random time and begin at site A (the first site in the first cycle) at that time. The agent will wait for potentially eligible patients at that site until the first eligible patient agreed to enroll and enter the data for that person. The agent will then proceed to the next site in that cycle (site C) and enroll the first eligible patients from that site. On completion of that cycle the enrollment will proceed to the final site in that cycle, and then proceed similarly the next cycle.

The study will have a monthly target to ensure that patients are enrolled over the course of the year. When the monthly target has been reached, enrollment will pause until the next month. It will then resume with a random site and time for the next cycle, etc. This process should ensure that patients are enrolled over the course of the dengue season, making the cohort reasonably representative. It will also balance the workload for recruitment and subsequently for follow-up interviews.

### Implementation resources

- Senior personnel who can design and manage the study and who can do training
- The existing public health centers’ lab technicians who can do the applied testing
- Purchase 120 dengue duo lab test kits for US$1800 (120 tests kit at US$15 each based on prevailing prices in Semarang in 2022)

### Enrollment procedures

**Figure 6.**
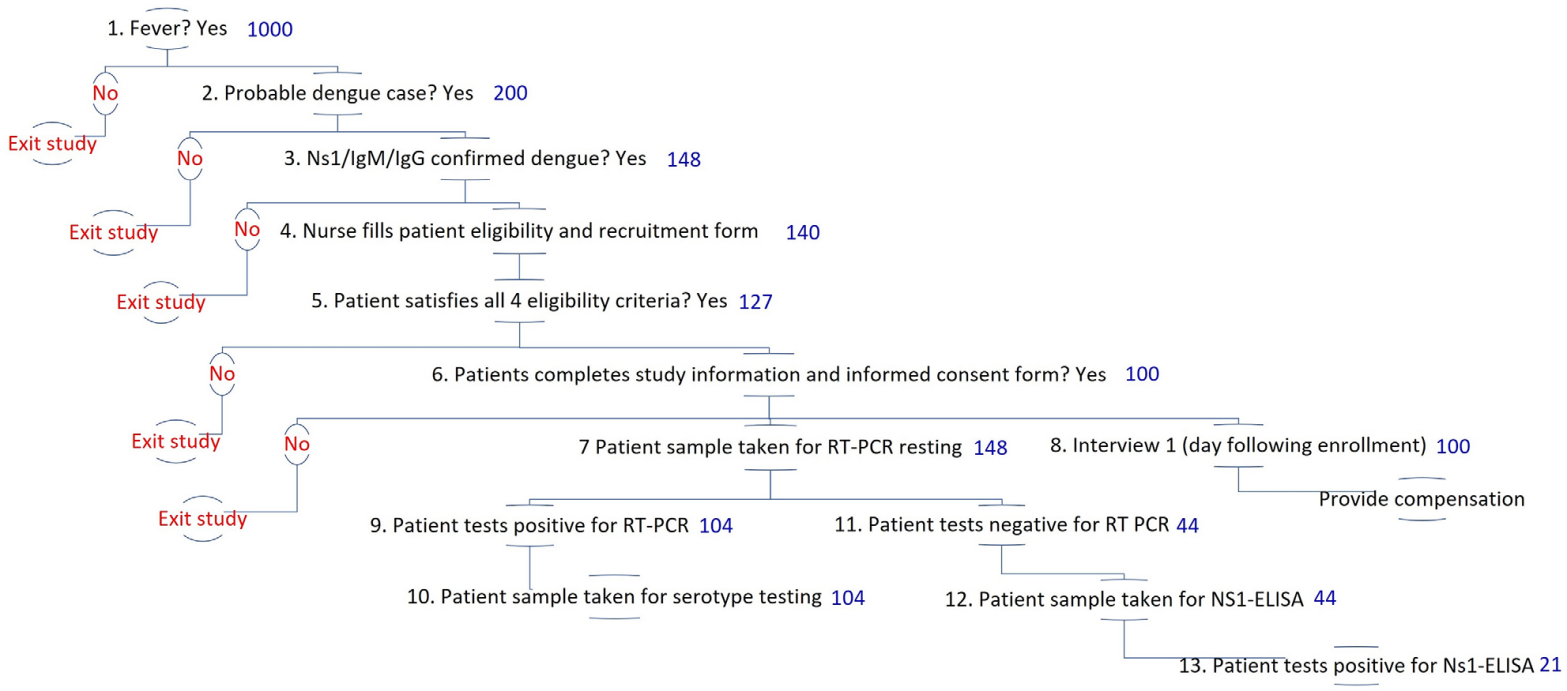
Illustrative patient enrollment and diagnostic testing process and estimations. Note: numbers displayed in blue indicate the number of patients expected at the beginning of each stage of patient enrollment and diagnostic testing, as described in detail in Table 4.

Figure 6 shows the planned enrollment and testing procedures. It describes the proportion of patients eligible at each stage in the sequence of diagnosing a suspected dengue test. These proportions are drawn from literature and the researchers’ prior experience ^45^.

By using the results of the NS1/IgM/IgG RDTs in the enrollment criteria for this study, the included patients will be those who may have experienced the onset of dengue symptoms within the past 3–5 days. NS1 is generally positive from the beginning of infection through day six. IgM is usually positive from day four to day 90 ^46^.

***Patients’ inclusion and exclusion criteria.*** Eligibility for patient enrollment requires meeting all of the following 4 criteria:

1. The patient has confirmed dengue by treating clinician, based on the diagnosis of a probable dengue infection (as described above), **and** tested positive for at least one of the additional NS-1 Ag and IgM tests included in the NS1/IgM/IgG RDT;
2. The patient plans to be available for providing follow-up information for six months after the dengue test (either in-person or by phone);
3. The patient (if 18 years of age or above) or caretaker (if the patient is 17 years of age or below) understands and can respond to normal questions;
4. The patient or caretaker provides appropriate Informed Consent. For adolescent patients aged 15-17, the patient must give Assent to confirm their desire to participate.

All patients who fail to satisfy one or more of the above eligibility criteria should be excluded from participation. Patients who test negative on both NS-1 and IgM but test positive on IgG tests will also be excluded, to rule out cases that likely do not have a current dengue infection but likely experienced a past infection. There is no age restriction for patient enrollment; rather, participation eligibility will be based on suspected dengue. Researchers should count the demographic categories of patients who declined to be tested or enter the study. This information will allow the researchers to perform weighting for under-represented groups.

***Incentives.*** We suggest the study offer an incentive of about one day’s minimum wage for each in-person interview and about a half of a day’s minimum wage for each telephone interview to compensate patients for their time and inconvenience. As of 2023 in Semarang, these amounts are about US$8 and US$4, respectively. If travel expense is substantial, that should be added for each in-person interview.

***Data collection.*** Data for this surveillance should be collected through recording of results from dengue-specific diagnostic testing and a series of patient interviews, as well as through supplemental facility-level data, including data on health facility costs.

## Data analysis regarding costs per episode

### Data collection data sources

In addition to the data obtained from patient interviews and diagnostic testing, information will be collected on the health facilities included in the method, including information needed to determine costing. This information will be gathered by team members from participating health facilities and input into a separate facility database ( Table 3). The facility database will be linked to the patient database and will include information on the facility name, ownership, type, location, and contact information of relevant facility representatives in the event the team needs to follow up on data or contact the health facility to resolve any other issues (see Figure 5) (see Table 3).

**Table 3.** Components of facility database to be created.

Component	Data included	Interval recorded
Facility identifier	Facility name	Two times
Facility ownership	Public vs. Private and ownership	Two times
Facility type	Hospital, *puskesmas,* clinic, and level	Two times
Facility location	Facility address	Two times
Facility contacts information	Name, position, email, and phone number of contact	Two times
Costing Data	Number of registered beds (where applicable), occupancy rate or the average census (number of patients per night), the annual number of outpatient visits, count of outpatient visits by day of the week, facility operating costs	Two times

**Table 4.** Description of patient enrollment and diagnostic testing proportions.

Step number	Step	Number of patients expected	Description	Scientific source
**1**	Fever	1,000	Targeted febrile cases over course of study, achieved by iteratively adjusting recruitment schedule (days of the week, dengue season).	Historic surveillance data from Semarang City 2011-2020. Utama and colleagues (2019) ^47^ estimate that the dengue share among febrile illnesses is in the range of 5% to 30% based on data from 2013 to 2016.
**2**	Probable dengue	200	Expected number of cases to be tested with NS1/IgM/IgG. The expected share of dengue cases among other febrile illnesses is 10-20%.	20% of step 1 based on Utama and colleagues (2019) ^47^.
**3**	Confirmed dengue (NS1/IgM/IgG RDT positive)	148	The expected %age of probable dengue cases with NS1/IgM/ IgG RDTs positive is 74%.	Step 2 multiplied by 74% based on the first author’s unpublished data from Sleman.
**4**	Nurses fill the recruitment form	1,125	The expected percentage of the completed form is 95% of the number of patients with NS1/IgM/ IgG RDT positive results.	95% of step 3 based on Sleman study
**5**	Patients satisfy eligibility criteria	127	The expected percentage of patients that satisfy the eligibility criteria is 90% of the number of completed recruitment forms.	90% of step 4 based on Sleman study
**6**	Completed study information and consent	810	The expected %age of patients with informed consent is 80% of the number of patients satisfying the eligibility criteria.	80% of step 5 is based on the Sleman study. This figure will allow us to follow up with those patients as our cohort participants.
**7**	RT-PCR testing	1,184	Includes all who satisfy step 3.	See step 3. We will confirm the patients’ who will be NS1/IgM/IgG RDTs positive with RT-PCR
**8**	Interview 1	810	Includes all those who satisfy steps 1-6.	See step 6.
**9, 13**	Virologically Confirmed Dengue (VCD)	1,006	The expected percentage of virologically confirmed dengue cases (RT-PCR or NS-1 ELISA) is 85% of the number of patients with NS1/IgM/IgG RDT positive results.	Nealon and colleagues (2016) estimate that 85% of clinically diagnosed dengue cases were virologically confirmed. ^35^
**9**	PCR +	835	The expected percentage of dengue RT-PCR positive cases among all VCD is 83%.	Utarini and colleagues (2021) ^17^ estimate that among all VCD, the share of RT-PCR positive cases was 83%
**10**	PCR serotyping	835	RT-PCR positive will be followed up with PCR serotyping.	Includes all expected RT-PCR positive cases
**11**	PCR negative	349	The expected percentage of dengue RT-PCR negative cases is 29.5% among those tested.	Calculated as the number of patients to receive RT-PCR testing (step 7) minus the number of expected RT-PCR positive (step 9)
**12**	NS-1 ELISA testing	349	Includes all those who satisfy step 11.	Calculated as the number of patients to receive RT-PCR testing (step 7) minus the number of expected RT-PCR positive (step 9)
**13**	NS-1 ELISA positive	171	The expected % age of NS-1 ELISA positive cases among VCD are 17%.	Step 9 multiplied by 17% based on Utarini and colleagues (2021) ^17^ estimate that among all VCD, the share of NS-1 ELISA positive cases was 17%.

The facility database will also include information on costing, using a macro-costing tool developed by the team. Data that are expected to be included in the facility database are described in Table 3. Data will be gathered either from the health facility informatics system, where applicable, or if this information is not electronically recorded, will be gathered directly from patient registers at participating health facilities. A unique benefit of this will be the inclusion and ability to generate data on multiple types of health facilities (public and private, as well as hospital and health center).

To capture additional data on the indirect and direct costs of a dengue episode from the patient’s perspective, the team will create a patient questionnaire with a set of standardized questions. These will capture out-of-pocket expenditures, direct non-medical cost, and indirect costs associated with a dengue episode. Combining these components will estimate the economic cost of dengue illness at the household level of the confirmed hospitalized and ambulatory cases. When entering data from patient questionnaires and hospital costing tools, data collectors will distinguish zero values from missing responses to allow all analyses to account for and address issues with missing data, such as using multiple imputation techniques.

### Data collection instruments

The final patient questionnaire will be designed to minimize the burden on both patients and researchers and will take about 20 minutes to complete. We suggest that researchers adapt existing instruments that have been validated for comparability with previous literature ^48^ and used in a similar context in Thailand. Patient questionnaires will include several short questions in the following areas:

***Questions on health-seeking behavior and expenditures.*** Questions will be asked to identify specific symptoms associated with DENV; their duration; health-seeking behavior related to the dengue episode, such as visits to a health clinic or pharmacy, and associated expenditures for medication and/or diagnostic tests. The first interview will capture information about the dengue episode since the onset of symptoms, while subsequent interviews will capture information since the last interview. Questions will include information such as:

a. How many visits with a medical professional has the patient had since (onset of symptoms or since the last interview) due to symptoms from the dengue episode?
b. How many nights has the patient spent at the hospital since (onset of symptoms or since the last interview) due to symptoms from the dengue episode?
c. How many days were you absent from work/school since (onset of symptoms or since the last interview) due to symptoms from the dengue episode?
d. What are the total out-of-pocket costs that the patient has spent on medical care, diagnostic tests, medications, and/or transportation for medical care since (onset of symptoms or since the last interview) due to symptoms from dengue episode?
e. Does the patient still have symptoms? If so, which symptoms? If not, when did symptoms stop?

***Ascertaining quality of life.*** The team will measure the impact of a complete symptomatic DENV episode (including both acute and persistent phases) on a patient’s quality of life (QoL) using an adapted Euro-Qol structured questionnaire (5-point Likert scale) ^49^ or similar tool. This tool will be adapted to capture the quality-of-life measurements at the time of patient interview and/or for a reference period (either since the onset of symptoms or since the previous interview).

***Ascertaining presenteeism.*** Questions will be asked to derive work productivity losses due to presenteeism. Possible validated tools that have all been widely used in other studies may include the WHO Health and Work Performance Questionnaire, which is a self-report instrument with measures for health-related productive costs associated with reduced work performance, sickness, absence, and work-related injuries; the Work Limitations Questionnaire (WLQ), which has frequently been used to measure impacts of chronic diseases and asks patients to rate their ability to perform various work demands (time, physical, mental, output); and the Stanford Presenteeism Scale (SPS-6), which is a shortened 6-question tool based on the original 36-item questionnaire (SPS-36) and includes self-reported measurements of health status and employee productivity.

***Macro-costing tool.*** Additionally, a macro-costing tool will be developed and used to create a facility database, linked to the patient database. Data derived from the macro-costing tool will be used to estimate the cost of a bed-day, a typical admission, and a typical outpatient service in public and private health facilities in the selected sites. The needed data for hospitals consists of the number of registered beds and occupancy rate or the average census (number of patients per night), the annual number of outpatient visits, and the hospital’s operating cost. The team will divide the hospitals into four categories based on ownership: national-public hospitals, district-public hospitals, non-governmental organization (NGO) hospitals, and private hospitals. For the primary health centers, including puskesmas and private clinics, researchers will use the average cost of providing personal health care services at the health centers (excluding public health services) using aggregated utilization and operational costs from the public health centers and the private clinics.

### Data analysis on dengue costs

Costs of each dengue episode will be derived from the patient interviews, surveillance data, macro-costing based on data reported by health facilities as well as national health insurance data from the selected sites, existing estimates of economic costs for the country in which the method will be implemented, and WHO-CHOICE estimate included in the internationally recognized OneHealth Tool. As performed in other costing studies, the team will value years of life lost based upon each country’s GDP per capita, minimum wage, wages of employed respondents, and unemployment rates. The team will employ a 3% annual discount rate, using the human capital approach. To incorporate lost days of school, we suggest that the team use an estimate of public expenditures incurred to value that day of school, as done previously ^31, 50^. We will estimate the indirect costs associated with a complete dengue episode, including productivity losses from work absenteeism and presenteeism. For presenteeism, we will input economic costs related to outpatient and inpatient visits derived from previous economic literature on dengue illness ^2^.

## Potential extensions to other cities

This method could be replicated in other cities that are also interested in informing their policies on control interventions. If one wanted national data, then a sample of multiple cities will be chosen that strike a balance between size and diversity in incidence rates of reported dengue.

## Ethical approval and consent

This illustration for Semarang City is based entirely on publicly available surveillance data. These are aggregate data with no person-level identifiers. Therefore, this paper’s focus (method development and simulation) does not fall under the purview of human studies research and no ethical approval was necessary nor obtained. However, a researcher seeking to apply this method will be identifying and interviewing individual patients and should seek guidance from an appropriate ethical committee at that stage.

## Discussion

### Benefits

Our proposed method represents a significant advancement in the field of dengue surveillance and burden estimation. In this section, we will discuss several key aspects that make this method efficient, effective, and well-suited for obtaining comprehensive insights into dengue at the municipal level.

***Pragmatism.*** Our design has sought to achieve pragmatism by involving just a few representative facilities. Project staff will then regularly visit those facilities for interviews. Efficiency in staffing is crucial when conducting epidemiological studies, especially in resource-constrained settings. Our method has been designed to optimize staffing requirements. Enrolling and repeatedly interviewing 100 patients with laboratory-confirmed dengue from a pool of approximately 1,000 patients with clinical dengue ensures that the study needs are met without overburdening resources. If current workloads allow, existing public health staff might be asked to incorporate the interviews into their existing responsibilities. If such merging were not feasible, existing staff might be offered a supplemental payment for each completed. Either approach not only conserves valuable resources but also ensures that the study remains cost effective.

***Representative sample acquisition.*** Obtaining a representative sample of dengue patients is fundamental to the validity and generalizability of our findings. To address this, our selection process is carefully structured to ensure the inclusion of a diverse range of cases, regardless of whether the year experiences a high or low number of dengue cases. This adaptability is critical for capturing the true burden of dengue within the municipality and enhancing the robustness of our results.

***Sequential enrollment across sites.*** We have implemented a sequential enrollment process across multiple sites, building on our prior experience in Mexico, Indonesia, and Thailand ^45^. This approach streamlines data collection and optimizes staffing allocation. By efficiently coordinating enrollment across sites, we ensure that our data collection process remains manageable and minimizes potential bottlenecks in participant recruitment.

***Leveraging prior research and collaboration.*** Our method benefits from the foundation laid by previous studies such as the ongoing study in Thailand ^51^ and the completed one in Yogyakarta ^19, 31^. By incorporating relevant questions from these studies, we enhance the depth of our data collection and ensure alignment with existing research efforts. Additionally, our collaborative development process involving external university researchers, local university researchers, and municipal health department managers in Semarang, fosters credibility and trust among local officials. This credibility is essential for ensuring that the results from this method can be confidently used to inform local policy decisions.

***Comprehensive assessment of dengue burden.*** In conclusion, while many surveillance studies focus solely on the number of dengue cases, our method offers a holistic approach. By capturing acute and chronic effects related to disease, economic burden, and psychological impacts (including presenteeism), we provide a comprehensive picture of the dengue burden. This comprehensive assessment is invaluable to the health system, payers, households, and local policymakers, as it goes beyond mere case counts and delves into the multifaceted dimensions of dengue’s impact on society.

### Dissemination

The main purpose of this study is to inform policy makers about the burden of dengue in their city. This information will inform their decision around dengue control intervention. At the start of the study, researchers should identify who the policy makers are, the type of information they largely require and the way that policy makers would use that information. Researchers should share those interpretations with the policy makers that they have identified and seek agreement. If there are differences, the researchers and policy makers should discuss them until disagreements are resolved. Resolution may entail revision to the study design and/or clarification of points initially misunderstood.

As the study proceeds, progress in terms of findings could be shared with policy makers. Finally, when the study is completed, outcomes and their interpretation should be shared in both presentations and written reports. Ample opportunity should be provided to policy makers to ensure that they understand the study, its implications, strengths and limitations.

Once explained and understood by local policy makers, findings should be shared with policy makers at higher levels (provinces and the national government). Finally, results should be shared with a broader scientific community through conference presentations and peer reviewed publications.

The investigators should create an anonymous file of the data that they have collected to ensure that individual participants are not identified. The anonymous file should remove not only names, medical record numbers, id numbers, but also the other information that could breach confidentiality, for example, include the name of the health facilities visited and the specific dates, and client’s precise demographics information (e.g., exact age and gender). Instead, researchers could supply broader descriptive information (e.g., a health center in the northern part of the city, or data collected during the month of January, respondent aged 50 - 59). Researchers should create a process for data access. The process should include confirmation by the researchers and the legal representatives of their organization that they will not try to identify individual respondents nor disseminate individual information. They should then create a process for secure data transfer.

### Limitations

While our method offers many advantages, it is essential to acknowledge a significant limitation. The enrollment process we have outlined, involving the sequential selection of 100 laboratory-confirmed dengue patients from a pool of approximately 1,000 clinical dengue cases, has not yet been tested in real-world practice. Although we have drawn from our experiences and previous research to design this process efficiently, its effectiveness in a practical setting remains unverified. Therefore, future implementation and validation of this enrollment approach are warranted to confirm its feasibility and reliability in diverse municipal contexts.

## Data Availability

No data are associated with this study. The surveillance data for developing this method are available from the City of Semarang Health Department ^41^.
