# Supplementary material for: Estimating dengue disease and economic burden to inform municipal-level policymakers: Method for a pragmatic city-level observational cohort study
Source: Gates Open Res. 2024 Aug 8;8:3. Originally published 2024 Jan 8. [Version 3] doi: 10.12688/gatesopenres.15015.3 (PMC11362393; doi:10.12688/gatesopenres.15015.3)
Supplement: Peer review history

# Reviewer report: Reviewer response for version 3

**DOI:** 10.21956/gatesopenres.17671.r37701

**Published:** 2024-08-29

**Authors:** Michael Tong

**Affiliations:** 1 The Australian National University (Ringgold ID: 2219), Canberra, Australian Capital Territory, Australia

The manuscript has been improved and addresses my concerns.

Is the rationale for developing the new method (or application) clearly explained?

Partly

Is the description of the method technically sound?

Partly

Are the conclusions about the method and its performance adequately supported by the findings presented in the article?

No

If any results are presented, are all the source data underlying the results available to ensure full reproducibility?

Partly

Are sufficient details provided to allow replication of the method development and its use by others?

No

Reviewer Expertise:

Infectious Diseases, Climate Change, Epidemiology

I confirm that I have read this submission and believe that I have an appropriate level of expertise to confirm that it is of an acceptable scientific standard.

# Reviewer report: Reviewer response for version 3

**DOI:** 10.21956/gatesopenres.17671.r37703

**Published:** 2024-08-27

**Authors:** David Roiz

**Affiliations:** 1 Univ. Montpellier, IRD, CNRS, Montpellier, France

The authors have made adequate amendments based on the comments.

Is the rationale for developing the new method (or application) clearly explained?

Yes

Is the description of the method technically sound?

Yes

Are the conclusions about the method and its performance adequately supported by the findings presented in the article?

Yes

If any results are presented, are all the source data underlying the results available to ensure full reproducibility?

Partly

Are sufficient details provided to allow replication of the method development and its use by others?

Yes

Reviewer Expertise:

Dengue transmission, economic costs, disease ecology

I confirm that I have read this submission and believe that I have an appropriate level of expertise to confirm that it is of an acceptable scientific standard.

# Reviewer report: Reviewer response for version 2

**DOI:** 10.21956/gatesopenres.17453.r37224

**Published:** 2024-07-19

**Authors:** David Roiz

**Affiliations:** 1 Univ. Montpellier, IRD, CNRS, Montpellier, France

The manuscript is very relevant for proposing an interesting episode-based methodology that highlights the impacts of the persistence dengue and presenteeism that are generally sub estimated and not appropriately evaluated on the dengue burden estimates.

 My main concern is the risk of dispersal due to an overambitious list of many  specific questions to be addressed, challenging the robustness and representative of the outputs of the study and their responses. A longitudinal study of 1.5 year duration and with a limited spatial sampling and limited number of enrolled patients cannot produce robust results about some of the questions. I suggest to the authors to reduce the number on question focusing on the ones that can be answered with a considerable confidence, representativeness and statistical significance. 

 Some minor comments are

 -Please correct the name to Butantan vaccine 

 -One reference of the cost-effectiveness of wMel is enough

 -the reference of Suaya is not included despite being cited

Is the rationale for developing the new method (or application) clearly explained?

Yes

Is the description of the method technically sound?

Yes

Are the conclusions about the method and its performance adequately supported by the findings presented in the article?

Yes

If any results are presented, are all the source data underlying the results available to ensure full reproducibility?

Partly

Are sufficient details provided to allow replication of the method development and its use by others?

Yes

Reviewer Expertise:

Dengue transmission, economic costs, disease ecology

I confirm that I have read this submission and believe that I have an appropriate level of expertise to confirm that it is of an acceptable scientific standard, however I have significant reservations, as outlined above.

# Response

**Published:** 2024-08-05

**Authors:** Nandyan Wilastonegoro

**Affiliations:** Health Policy and Management, Universitas Gadjah Mada, Yogyakarta, Special Region of Yogyakarta, Indonesia

**Reviewer comment: **The reviewer commented the paper is very relevant for proposing an interesting episode-based methodology which highlights the impacts of the persistence dengue and presenteeism that are generally sub estimated and not appropriately evaluated on the dengue burden estimates.

**Author response: **Thank you

**​​​​​​​Reviewer comment: **The reviewer concerned about the risk of dispersal due to overambitious list of many specific questions to be addressed making it challenging to the robustness and representativeness of the outputs of the study and their responses. **​​​​​​​**

**Author response: **As noted in response to Michael Tong (reviewer 3), we have clarified that domain 1 is the core domain and others are optional.  We also expanded on the methods and supporting references.

**Reviewer comment: **The reviewer commented on the duration of 1.5 year with a limited spatial sampling and limited number of enrolled patients. The reviewer concerned it would not produce robust results on some of the questions. **​​​​​​​**

**Author response: **This reviewer has raised an important point.  We have expanded on the discussion between precision in a single year versus multiple years of study (see line 242 and following)

**Reviewer comment: **The reviewer suggested to reduce the questions focusing on answerable questions with considerable confidence, representativeness and statistical significance. **​​​​​​​**

**Author response: **As noted above, we noted that domain 1 is now the core and other domains are optional.

**Reviewer comment: **Another comment was asking to correct the name of Butantan vaccine.

**​​​​​​​Author response: **Corrected

**Reviewer comment: **The reviewer asked to add reference on wMel cost-effectiveness.

**​​​​​​​Author response: **We added the label wMel to the sentence with the citation to Brady et al (formerly reference 18).

**Reviewer comment: **A mention on Suaya paper citation, it was mentioned but not included in the reference.

**​​​​​​​Author response: **It has now been added.

**Reviewer comment: **The reviewers had not yet approved the paper

**Author response:** We have added citations and made numerous clarifications and corrections

# Reviewer report: Reviewer response for version 2

**DOI:** 10.21956/gatesopenres.17453.r37227

**Published:** 2024-07-19

**Authors:** Michael Tong

**Affiliations:** 1 The Australian National University (Ringgold ID: 2219), Canberra, Australian Capital Territory, Australia

The study is quite ambitious, addressing 13 questions across four domains: epidemiology, persistent dengue, quality of life, and economic burden. I am not sure this is a good idea to include all of these in one paper.

 Further, I am not sure whether the details of the method can be used to reproduce the results, particularly the data analysis for Domains 2,3, and 4. It might be better to focus on one domain at a time, providing detailed methods, data sources, and one or two examples to demonstrate the methodology.   

 Minor comments:

 Introduction – Dengue control technologies: could I ask where we can verify the Takeda vaccine (61.2% efficacious) and the newest Bhutantan vaccine (79.6%)? Any references or evidence?  

 Figure 1: Please add the country to the figure title.

 Table 1: Please explain the hospital level in the table caption.

Is the rationale for developing the new method (or application) clearly explained?

Partly

Is the description of the method technically sound?

Partly

Are the conclusions about the method and its performance adequately supported by the findings presented in the article?

No

If any results are presented, are all the source data underlying the results available to ensure full reproducibility?

Partly

Are sufficient details provided to allow replication of the method development and its use by others?

No

Reviewer Expertise:

Infectious Diseases, Climate Change, Epidemiology

I confirm that I have read this submission and believe that I have an appropriate level of expertise to state that I do not consider it to be of an acceptable scientific standard, for reasons outlined above.

# Response

**Published:** 2024-08-04

**Authors:** Nandyan Wilastonegoro

**Affiliations:** Health Policy and Management, Universitas Gadjah Mada, Yogyakarta, Special Region of Yogyakarta, Indonesia

**Reviewer comment: **The reviewer commented on 13 questions over four domains is ambitious. The reviewer suggested not to include all of four domains in one paper.

**Author response:** We have revised the text to indicate that domain 1 is the core domain.  We suggest that this one be addressed by all users.  We have clarified that domains 2, 3 and 4 are optional supplemental components.  They do not necessarily need to be included.  They should be selected based on the importance of the information to policymakers and the resources available.

**Reviewer comment: **The reviewer doubted the details of the method can be used to reproduce results, particularly data analysis for domain 2, 3, and 4.

**Author response: **We have expanded on the methods and added additional citations.  We also noted above that these components are optional, so a user can adapt or omit them as needed.

**Reviewer comment: **The reviewer suggested to focus on one domain at one time, for example providing detailed method, data sources, and one or two examples to demonstrate the methodology.

**​​​​​​​Author response: **We appreciated this constructive suggestion.  Under domain 1, we noted that our methods are an extension of an approach we previously applied in Yogyakarta and we provided the citation for background (see reference 31, Cost of dengue illness in Indonesia across hospital, ambulatory, and not medically attended settings.)  Based on that prior work and other experience, we have added an example to illustrate each of the domains.

**Reviewer comment: **The reviewer asked references or evidence which verify Takeda vaccine efficacy (61.2%) and Butantan vaccine efficacy (79.6%)?

**​​​​​​​Author response: **We have added the source – Thomas et al 2023.

**Reviewer comment: **The reviewer commented on Figure 1, asking to add the country to the figure title.

**​​​​​​​Author response: **This was done.

**Reviewer comment: **Another comment on Table 1, the reviewer asked to explain hospital level in the table caption.

**​​​​​​​Author response: **An explanatory paragraph was added.

**Reviewer comment: **The reviewers had not yet approved the paper

 ​​​​​​​ **​​​​​​​Author response: **We have added citations and made numerous clarifications and corrections

# Reviewer report: Reviewer response for version 1

**DOI:** 10.21956/gatesopenres.16348.r36005

**Published:** 2024-05-11

**Authors:** Fardhiasih Dwi Astuti

**Affiliations:** 1 Ahmad Dahlan University, Yogyakarta, Indonesia

The title and the abstract are to be aligned. Abstracts still need to present results. The abstract must consist of background, methods, results, and conclusions.

 The purpose of the research needs to be clarified. In the background, there are many research questions, but how to answer all the research questions needs to be explained using scientific methods. Write down the purpose of the research.

 It is essential to clarify the purpose of the research. While many research questions may be in the background, explaining how they will be answered using scientific methods is essential. Therefore, it is crucial to write down the purpose of the research.

 The research methods used must be explained in detail. This includes the research design, how samples were collected, inclusion and exclusion criteria used, and how each variable was assessed and analyzed. Finally, it is essential to explain the findings of the research.

 It is recommended that the manuscript follow the journal guidelines.

Is the rationale for developing the new method (or application) clearly explained?

No

Is the description of the method technically sound?

Partly

Are the conclusions about the method and its performance adequately supported by the findings presented in the article?

No

If any results are presented, are all the source data underlying the results available to ensure full reproducibility?

No

Are sufficient details provided to allow replication of the method development and its use by others?

No

Reviewer Expertise:

vector born disease and public health

I confirm that I have read this submission and believe that I have an appropriate level of expertise to state that I do not consider it to be of an acceptable scientific standard, for reasons outlined above.

# Response

**Published:** 2024-06-11

**Authors:** Nandyan Wilastonegoro

**Affiliations:** Health Policy and Management, Universitas Gadjah Mada, Yogyakarta, Special Region of Yogyakarta, Indonesia

1. The reviewer mentioned that our title did not align with the abstract
  - **Authors' response:** We revised the background section of our abstract to better align with the title, highlight the method nature of our study.
2. The reviewer asked us to clarify and clearly state the purpose of our study
  - **Authors' response: **We added a subheading at line 185 as follows: “Overall purpose of study,”  and clarified some of the text in that section.
3. The reviewer required us to detail how we would solve the listed research questions
  - **Authors' response: **To clarify, we added the word “Domain” around each of our areas.  In the subsequent text, we, inserted sub-headings for each of our four domains to clarify the part of our methods that respond to each of our research questions
4. The reviewer asked us to clearly state the purpose of the study
  - **Authors' response: **See our response to the preceding comments.
5. The reviewer asked us to detail our method i.e., study design, sample collection, inclusion and exclusion criteria, and analysis
  - **Authors' response: **The introduction to Domain 1 indicates explicitly that our overall design is multi-stage observational study.  The section “Inclusion and exclusion criteria” around line 413 addresses these factors.  We created a section called “Data analysis” around line 537 (just before “Ethical considerations”).
6. Another mention was about discussing our findings.
  - **Authors' response: **As this paper is purely a methods paper, we do not have any findings to discuss.  We did, however, add a recommendation for users to disseminate their findings and explain that further in the section entitled “Dissemination.”

# Reviewer report: Reviewer response for version 1

**DOI:** 10.21956/gatesopenres.16348.r36009

**Published:** 2024-04-03

**Authors:** Chanh Ho Quang

**Affiliations:** 1 Dengue, Oxford University Clinical Research Unit, Ho Chi Minh City, Vietnam; 2 Oxford University Clinical Research, Ho Chi Minh City, Vietnam

The study protocol aims to address several gaps in the dengue field. This comprehensive approach would provide meaningful evidence to inform local and global stakeholders to tackle this global public health disease. However, the protocol is quite vague which may not allow for reproducibility. The operation through local public and private healthcare system seems complicated which may produce the selection bias considering a relatively small sample size. It is also unclear how authors would account for disease severity, age, sex, etc.  as it would affect patient's experience and potential sequela. Furthermore, there is no good rationale for the chosen sample size.  As seasonality would affect the monthly number case, the recruitment target needs to weight for this aspect to provide a representative population. There are a number of questionnaires/tools mentioned but the lack information about validating these tools. Also, there is no strategy to retain participants. Personally, I think it would make more sense to quantify education-day lost as children is still the main  population of this disease. 

 Another point is about ethical approval, there is no enough information about the ethical approval process and how the informed consent/assent form will be obtained. 

 There is no result in this manuscript.

Is the rationale for developing the new method (or application) clearly explained?

Partly

Is the description of the method technically sound?

Partly

Are the conclusions about the method and its performance adequately supported by the findings presented in the article?

No

If any results are presented, are all the source data underlying the results available to ensure full reproducibility?

No source data required

Are sufficient details provided to allow replication of the method development and its use by others?

No

Reviewer Expertise:

dengue, clinical study

I confirm that I have read this submission and believe that I have an appropriate level of expertise to confirm that it is of an acceptable scientific standard, however I have significant reservations, as outlined above.

# Response

**Published:** 2024-06-11

**Authors:** Nandyan Wilastonegoro

**Affiliations:** Health Policy and Management, Universitas Gadjah Mada, Yogyakarta, Special Region of Yogyakarta, Indonesia

1. The reviewer questioned our publication whether it could be reproducible or not.
  - **Authors' response:** We added further details about our selection procedures and concluded (around line 450): “These operational details should make our results reproducible.”
2. The reviewer highlighted potential selection bias due to a relatively small sample size.
  - **Authors' response:** Our design seeks to minimize bias by selecting sites across multiple strata (facilities both with and without inpatient care).  Given the purpose of the study, we acknowledge that it seeks to be pragmatic rather to give timely data at an affordable cost.
3. The reviewer mentioned the paper had no good rationale for the chosen sample size.
  - **Authors' response:** Economic data per episode tend to be more stable (less variable) than epidemiologic data, so smaller sample sizes can be sufficient for economic studies compared to epidemiologic studies.  We added a paragraph beginning around line 233 about sample size.  We show that the proposed sample size can generate a key economic result (overall cost per hospitalized episode) with an acceptable accuracy of 10%. 
4. The reviewer required us to weigh the recruitment target anticipating seasonality.
  - **Authors' response:** We mentioned in our manuscript that this method includes one full dengue season and one partial dengue season in the first year (p5). We also stated how we randomly select cases to invite into the study through creating cycles and putting sites in a random order. This process ensures patients are enrolled over the course of the dengue season to make the cohort reasonably representative (p11).
5. The reviewer asked on how we would validate questionnaire/tools
  - **Authors' response:** We mentioned that we would recommend applying the method using standardized questionnaire designed to minimize burden of both patients and researchers. We suggested selecting items from existing validated instruments (p14). For example:
    - Ascertaining quality of life using Euro-Qol structured questionnaire (p14)
    - Ascertaining presenteeism using Work Performance Questionnaire, Work Limitations Questionnaire (WLQ)
  - The use of validated items saves time, increases confidence in our results, and ensures comparability with previous findings.
6. The reviewer asked was about our strategy to retain participants.
  - **Authors' response:** We mentioned in Figure 6 how we would recruit and retain participants. For example, we will offer refreshments to participants each time they come to the facility for study purposes.
7. The reviewer noted that we did not write specific results.
  - **Authors' response:** As our paper is entirely a methods paper, we do not have any results. 
